# Editorial: Differences in Pain Biology, Perception, and Coping Strategies: Towards Sex and Gender Specific Treatments

**DOI:** 10.3389/fnins.2021.697285

**Published:** 2021-06-18

**Authors:** Parisa Gazerani, Anna Maria Aloisi, Hiroshi Ueda

**Affiliations:** ^1^Department of Life Sciences and Health, Faculty of Health Sciences, Oslo Metropolitan University, Oslo, Norway; ^2^Department of Health Science and Technology, Faculty of Medicine, Aalborg University, Aalborg, Denmark; ^3^Department of Medicine, Surgery and Neuroscience, University of Siena, Siena, Italy; ^4^Department of Pharmacology and Therapeutic Innovation, Nagasaki University Institute of Biomedical Sciences, Nagasaki, Japan; ^5^Department of Molecular Pharmacology, Kyoto University Graduate School of Pharmaceutical Sciences, Kyoto, Japan

**Keywords:** sex, pain, gender, pain perception, pain biology, pain treatement

Sex and gender differences in pain have been a point of focus in the medical field and continue to gather increasing interest (Dance, 2019; Mogil, 2020; Templeton, 2020). In this line, biological, psychological, and social influences on pain have been investigated (Fillingim, 2015; Keogh, 2020). Building upon the accumulating knowledge of mechanisms underlying sex and gender differences in pain (Bartley and Fillingim, 2013; Chen et al., 2018; Kim and Kim, 2020) has led to an escalating interest in gender and sex-specific treatments in medicine and pain (Packiasabapathy and Sadhasivam, 2018; Mauvais-Jarvis et al., 2020; Templeton, 2020). This field is gradually advancing toward identifying ways to apply available knowledge of sex and gender-related differences for pain treatments (Planelles et al., 2020; Rathbone et al., 2020). It is expected that the future will witness more novel treatment strategies and prevention with a focus on sex and gender-based treatment of pain.

This Research Topic (https://www.frontiersin.org/research-topics/10654/differences-in-pain-biology-perception-and-coping-strategies-toward-sex-and-gender-specific-treatme) intended to collect current evidence and encourage further research to highlight the value and importance of sex and gender-based understanding and treatments for pain and how current knowledge can be implemented into clinical practice. We present six articles that cover both basic and clinical studies to provide a broad spectrum of opportunities and gaps in the field and perspective for the future. The manuscripts provide a better understanding of why females and males are different in pain processing, and how such differences can affect treatment strategies.

Zin et al. presented that adherence to opioid therapy depends on age and gender, where younger patients seem to be less adherent to opioid use. It was also demonstrated that male patients using opioids for pain was associated with less adherence. These findings highlight that attitude toward opioid use is different between females and males and that a monitoring system might assist the users, based on their sex and age, differently. Literature also shows that younger individuals might be at risk for misuse of opioids (Hudgins et al., 2019) and older individuals might be at risk of cognitive and functional impairments (Pask et al., 2020), which both warn about suboptimal adherence and the importance of considering elements such as age and sex in pain therapy. Knowing important factors that might contribute to poor adherence (Mathes et al., 2014; Gast and Mathes, 2019) would help in better planning and preventing potential risks, for example, patient education, health care providers' attention, and appropriate monitoring plans in opioid therapy. In line with considering patient age and sex in therapeutic plans for pain, Gazerani and Cairns reviewed the literature for sex-based treatment potential for migraine, which is a known sexually dimorphic disorder with a higher prevalence in women (Al-Hassany et al., 2020; Ashina, 2020). Although the literature was less active in reporting sexually distinct responses to abortive (for example triptans) and prophylactic pharmacotherapy (beta-blockers, calcium channel blockers, and selective serotonin reuptake inhibitors) for migraine, both basic research findings and scattered clinical cases present that sex-based responses exist and that the differences might be due to several factors (including pharmacokinetics and pharmacodynamics; Soldin and Mattison, 2009; Soldin et al., 2011). This review (Gazerani and Cairns) highlights the value of future study designs for including sex differences in analgesic effects and side effects, as well as contributions from sex hormones, genetic variation, and co-administration of drugs for comorbid conditions, such as depression (Fillingim, 2002; Packiasabapathy and Sadhasivam, 2018). Psychosocial factors (Keogh, 2013) (such as expectations, stereotypes, cultural differences, pain-related beliefs, past experiences of pain, and environmental stress) seem equally important for the next studies. It is known that coping strategies, in general, are different among genders; for example, women apply coping strategies such as social support, emotion-focused techniques, attention focus, cognitive re-interpretation, and positive self-statement, while problem-focused techniques and behavioral distraction are more common strategies used by men (El-Shormilisy et al., 2015). Related to orofacial pain, Liu et al. presented that an analgesic effect can be achieved through peripheral opioid receptors and endogenous opioid peptides and therapeutic agents that can act within peripheral orofacial tissues might offer novel therapeutic options that can limit central nervous system side effects (Machelska and Celik, 2018). Receptor expression and sex-based differences have been found, for example, in peripheral mu-opioid receptor in rat orofacial persistent pain model (Bai et al., 2015), sexual dimorphism in kappa opioid receptors in the rat temporomandibular joint (Clemente et al., 2004), and sex differences in the contribution of potassium channels in trigeminal ganglia (Niu et al., 2011). Hence, further exploration is encouraged as to whether and how sex-related responses could help in the identification of optimal treatment plans for orofacial pain in either sex. Exploration can be done in animal models of pain or human experimental models of pain. Ceccarelli et al. investigated β-caryophyllene (BCP), which is a plant-derived compound and full agonist of cannabinoid receptor B2 (CB2), in the formalin inflammatory model of pain. A sex difference was found in the BCP-induced analgesic effect, wherein males' BCP showed a greater analgesic effect. This can be due to a heterogeneity in cannabinoid system and mRNA levels of CB1 and CB2 in animal brains of either sex (Craft et al., 2013). Evidence supports that the endocannabinoid system is strongly influenced by circulating levels of estradiol (Santoro et al., 2021). Interestingly, when the estradiol levels are higher, the cannabinoid system is more effective in females (Brents, 2016). In the Ceccarelli et al. study, females were examined in the estrus phase, which is accompanied by very low levels of estrogens, which can explain the lower BCP-induced analgesic effect in females. Collectively, findings from this study highlight the importance of considering the sex of animals for testing the analgesic efficacy of novel compounds. This can guide dosing, interval, and potential effects or side effects in translational studies to humans.

Ueda et al. investigated if a sex difference existed in mechanical allodynia and hypersensitivity to electrical stimulation in a model of generalized pain that mimics some aspects of fibromyalgia (Sluka and Clauw, 2016). The study by Ueda et al. applied several methods to provide evidence on sexual dimorphic mechanisms underlying pain hypersensitivity. An interesting finding was the identification of the involvement of microglia. The repeated brain treatments with minocycline prevented acid-induced generalized pain (AcGP) in male, but not female, mice. The allodynia was reproduced in naïve mice by the intravenous administration of splenocytes derived from both male and female AcGP mice, but female splenocytes showed more prolonged allodynia. CD4-positive T cells derived from splenocytes showed equipotent allodynia, being consistent with clinical studies with fibromyalgia patients (Banfi et al., 2020). This study (Ueda et al.) not only presents that mechanisms underlying pain and hypersensitivity are different in females and males, but that response to treatment could dramatically be affected by sex, and the contribution of non-neuronal cells, such as microglia, is highly important. Presentation of hypersensitivity and sex-related features has also been presented in a human experimental model of pain (Racine et al., 2012a,b). Ferentzi et al. demonstrated that young men had significantly higher pain threshold and tolerance levels compared with females, whereas cardiac interoceptive accuracy was not associated with pain sensitivity. This study highlights the value of considering sex in human experimental models and how the responsiveness might exert different responses. Further studies can explore potential mechanisms underlying such differences between males and females. Besides, since this study only focused on participants of a young age, further investigation can add age factor into the interaction. Knowledge from elements of pain features can help in understanding responsiveness to pain therapy when similar techniques are used for the analgesic effect of tested compounds in clinical trials. It is highly important to consider sex-related differences when findings are translated from animals to humans and if any differences exist across species (Smith, 2019).

Collectively, this special issue provides the state-of-the-art in the fascinating area of sexual dimorphism in pain features and pain treatment. We are grateful to all authors for their enthusiasm and expert contribution in covering the topic from basic to clinical research, highlighting potential mechanisms, and how current findings can guide consideration of gender, sex, and age and their interactions in pain research and treatment. We also appreciate the full support of Frontiers for their exceptional editorial contribution throughout the process. We hope to raise awareness, stimulate curiosity, highlight the value, potential, and limitations, and contribute a deeper understanding of the fundamentals for sex-based research and treatment in pain.

## Author Contributions

PG collected the manuscripts and wrote the first draft of the editorial. AA and HU reviewed and commented on revisions. All authors approved the final version for submission.

## Conflict of Interest

The authors declare that the research was conducted in the absence of any commercial or financial relationships that could be construed as a potential conflict of interest.
